# Detection of Four Novel Polymorphisms in PrP gene of Pakistani sheep (Damani and Hashtnagri) and goats (Kamori and Local Hairy) breeds

**DOI:** 10.1186/1743-422X-8-246

**Published:** 2011-05-20

**Authors:** Amjed Hussain, Masroor E Babar, Muhammad Imran, Ikram U Haq, Muhammad M Javed

**Affiliations:** 1 Government College University, Lahore 54000, Pakistan; 2 University of Veterinary and Animal Sciences, Lahore 54000, Pakistan; 3 Centre for Research in Endocrinology and Reproductive Sciences, University of Health Sciences (UHS), Khayaban-e-Jamia Punjab, Lahore 54600, Pakistan

## Abstract

Scrapie is a fatal neurodegenerative disorder of sheep and goats caused by post-translational conformational change in the host-encoded prion protein (PrP^C^). Susceptibility or resistance to scrapie has been associated with the presence of polymorphisms in the prion protein (PrP) gene. In the present study, we analyzed the PrP gene sequence to determine the frequency of polymorphisms in 56 sheep (28 each from Damani and Hashtnagri breeds) and 56 goats (28 each from Kamori and Local Hairy breeds). A total of 7 amino acid polymorphisms were detected in the PrP gene for sheep and 4 for goats. These amino acid polymorphisms were combined in 13 alleles and 15 genotypes in sheep and 5 alleles and 6 genotypes in goats. The overall frequency of the most sheep scrapie-resistant polymorphism (Q171R) was calculated to be 0.107. The most scrapie-susceptible polymorphism (A136V) was not detected in any of the studied sheep. The overall frequency of scrapie-associated polymorphism (H143R) in goats was found to be 0.152. Along with already known amino acid polymorphisms, two novel polymorphisms were also detected for each of sheep (Q171N and T191I) and goats (G22C and P63L). However, the overall frequency of these polymorphisms was extremely low.

## Introduction

Scrapie in sheep and goats is the most ancient form among a group of chronic neurodegenerative disorders cumulatively known as prion diseases or transmissible spongiform encephalopathies (TSEs). TSEs other than scrapie include Cruetzfeldt-Jacob disease in humans, chronic wasting disease in deer and elk, mad cow disease in cattle and transmissible mink encephalopathy [1]. The unconventional etiology of TSEs is evidently associated with the conversion of a normal host-encoded prion protein (PrP^C-Cellular^) to abnormal pathogenic conformers (PrP^Sc-Scrapie^) which get accumulated in the brain and lymphoid organs of cases in the form of fibrillar aggregates [2]. The detection of PrP^Sc ^in relevant tissues is therefore regarded as the major diagnostic marker for the clinical evaluation of TSEs [3]. However, the sequence analysis of the PrP gene can also be used as a preliminary test to infer about the presence of a TSE [4]. The major concerns about TSEs are multiple PrP^Sc ^strains causing variable long incubation periods depending on their infectious potential and the genetic makeup (species barrier) of the species involved [5,6] and the lack of suitable therapeutics [7,8], which have paced up the prion research all over the world, especially in Europe and Northern America.

The key factors controlling the onset or incidence of TSEs are the PrP amino acid sequence and the expression level of the PrP gene. Therefore, the use of the PrP genetics has been suggested to control and eventually eradicate TSEs, especially animal TSEs. After the establishment of the genetic association of ovine and caprine PrP polymorphisms with natural or typical scrapie susceptibility and the disease progression, the study of the allele frequencies of the major PrP variants in sheep and goats has attained much attention worldwide and huge efforts have been made in this regard especially in Europe, USA, China and Japan. Several amino acid polymorphisms related to scrapie susceptibility have been reported for both sheep and goats [9]. Sheep exposed to natural or experimental PrP^Sc ^infection have been shown to gain maximum scrapie resistance in the presence of Q171R polymorphism and maximum scrapie susceptibility in the presence of A136V polymorphism [10]. A three codon system based on A136V, R154H and Q171R/H polymorphisms has been characterized with five alleles (ARQ, VRQ, AHQ, ARR and ARH) which can be combined to form 15 genotypes i.e. ARR/ARR or VRQ/ARQ. These 15 genotypes have helped to develop a five-group risk classification system (R1-R5) which has been used in breeding and eradication programs [11,12]. The most resistant R1 genotype is ARR/ARR and the most susceptible R5 genotypes are VRQ/VRQ, VRQ/ARQ, VRQ/ARH and VRQ/AHQ. Though ovine PrP polymorphism has been abundantly linked to the scrapie risk, a few studies have been carried out for the analysis of caprine PrP gene in this regard. This is due to the fact that the prevalence of typical scrapie outbreaks in goat is comparatively much lower than in sheep. However, caprine PrP polymorphisms I142M, H143R, N146S/D, R154H, R211Q and Q222K have been shown to be associated with resistance or low scrapie risk [13-18] and are therefore being studied worldwide [19,20]. Many other ovine and caprine PrP polymorphisms have also been reported, which can be reviewed in [9,20-23].

Sheep and goats are major livestock species of Pakistan contributing almost 33% of the total red meat consumed in the country. These species also provide the basic economic source for many people dwelling in the underdeveloped parts of the country. The present study was conducted for the sequence analysis of coding sequences of the PrP gene in two each of Pakistani sheep (Damani and Hashtnagri) and goats (Kamori and Local Hairy) breeds. Although no scrapie case has been reported in any of Pakistani sheep and goats, knowing the PrP gene polymorphism spectrum in these species may be helpful for future breeding plans in terms of scrapie resistance.

## Materials and Methods

The study was approved by Ethical Review Committee of University of Veterinary and Animal Sciences Lahore. Twenty-eight animals from each of two sheep (Damani and Hashtnagri) and two goats (Kamori and Local Hairy) breeds were selected for blood sampling from their respective breeding tracts and Government Livestock Farms. Kamori was sampled from Sindh, Local Hairy from Punjab, and Damani and Hashtnagri from Khyber Pakhtunkhwa. The selection of more than one animal from the same pedigree was avoided to increase the genetic heterogeneity among the samples. The genetic heterogeneity was further increased by selecting only a few unrelated animals from a flock. Ten ml blood samples of selected animals were collected from their jugular vein into 50 ml falcon tubes, containing 100 μl of 0.5 M EDTA (pH 8.0), and immediately transferred to an ice box and stored at -20°C until further processing.

Extraction of genomic DNA from whole blood was performed by inorganic procedure [24]. A DNA fragment of 876 bp length containing the entire coding region in exon 3 of the PrP gene (GeneBank accession, DQ346682), was PCR-amplified by using a primer pair (F5'-1CTTTAAGTGATTTTTACGTGG21-3') and (R5'- 854TGGCAAAGATTAAGAAGATAATG876-3'). PCR reactions were carried out in a total volume of 25 μl containing 50 ng genomic DNA, 10 mM Tris/HCl (pH 8.3), 50 mM KCl, 1.5 mM MgCl_2, _200 μM dNTPs, 10 ρmoles of each primer and 1.5 U Taq polymerase (Fermentas Inc. USA). Thermal program of PCR was set as initial denaturation at 94°C for 4 min, followed by 35 cycles of denaturation at 94°C for 45 s, annealing at 54°C for 45 s and extension at 72°C for 1 min. A final 10 min extension at 72°C was also added to the program. The PCR products were purified by 64% ethanol at final concentration and were used in dideoxy sequencing reactions. Additional to above-mentioned primer pair, PrP7-U (5'- 369AGCAGTGGTAGGGGGCCTTGGT391- 3') and PrP-L2 (5'- 430TTTGGCTTACTGGGCTTGTTCC451- 3') primes were also used in sequencing PCR reactions for bidirectional sequencing of the entire coding region of the PrP gene. The amplified products of sequencing PCR were purified using 70% ethanol and resolved on an ABI 3130 capillary DNA analyzer (Applied Biosystems, Inc., Foster City, CA). The PrP gene sequences were visually inspected using the Chromas Lite (2.01) software and then transported to BLAST for their alignment with the standard sequence.

## Results

Sequencing of the PrP gene showed seven amino acid polymorphisms in two sheep breeds (Damani and Hashtnagri) and four in two goat breeds (Kamori and Local Hairy) of Pakistan (Table 1). These amino acid polymorphisms were combined in 13 alleles and 15 genotypes in sheep and 5 alleles and 6 genotypes in goats. The maximally detected allele in sheep with an overall frequency of 0.500 was of wild-type (G_127_R_151_Y_152_Q_171_Q_189_T_191_). The G_127_R_151_Y_152_Q_171_L_189_T_191 _allele was the second frequent allele in sheep with an overall frequency of 0.116. Ultimately the genotypes with these two alleles were detected with higher frequency. Two alleles G_127_R_151_Y_152_N_171_Q_189_T_191 _and G_127_R_151_Y_152_Q_171_Q_189_I_191_) carried the new amino acid polymorphisms Q171N (cag171aat) and T191I (aca191ata). These alleles were found only once (heterozygous- 1/56 = 0.009) in two different sheep. Additionally, two previously reported silent mutations (agg231cgg - R231R and ctc237ctg - L237L) were also detected in six animals.

**Table 1:** Allelic and genotypic frequencies of PrP in Damani and Hashtnagri, and Kamori and Local Hairy

	Allelic frequencies							
		**Sheep breeds**				**Goat breeds**		
				
		**Damani Hashtnagri**		**Both**		**Kamori**	**Local Hairy**	**Both**

		**n/56**	**n/56**	**n/112**		**n/56**	**n/56**	**n/112**

1	G_127_R_151_Y_152_Q_171_Q_189_T_191_	0.464	0.536	0.500	G_22_P_63_H_143_S_240_	0.232	0.375	0.304
2	SRYQQT	0.054	0.161	0.107	GPHP	0.589	0.464	0.527
3	SRYRQT	0.018	0.000	0.009	CPHP	0.018	0.000	0.009
4	SRYHQT	0.018	0.036	0.027	GLHP	0.018	0.000	0.009
5	SRYQLT	0.018	0.018	0.018	GPRP	0.143	0.161	0.152
6	GCYQQT	0.036	0.018	0.027				
7	GRPQQT	0.018	0.018	0.018				
8	GRYQQI	0.000	0.018	0.009				
9	GRYRQT	0.161	0.036	0.098				
10	GRYHQT	0.000	0.018	0.009				
11	GRYNQT	0.000	0.018	0.009				
12	GRYKQT	0.071	0.036	0.054				
13	GRYQLT	0.143	0.089	0.116				

	**Genotypic frequencies**							

		n/28	n/28	n/56	n/28	n/28	n/56	

1	GRYQQT/GRYQQT	0.357	0.464	0.411	GPHS/GPHS	0.107	0.286	0.196
2	SRYQQT/SRYQQT	0.00	0.107	0.054	GPHS/GPHP	0.250	0.179	0.214
3	SRYQQT/SRYRQT	0.036	0.00	0.018	GPHP/GPHP	0.286	0.214	0.250
4	SRYQQT/SRYHQT	0.036	0.071	0.054	CPHP/GPHP	0.036	0.00	0.018
5	SRYQQT/SRYQLT	0.036	0.036	0.036	GLHP/GPHP	0.036	0.00	0.018
6	GRYQQT/GCYQQ	0.071	0.036	0.054	GPRP/GPHP	0.286	0.32	0.304
7	GRPQQT/GRYKQ	0.036	0.036	0.036				
8	GRYQQT/GRYQQI	0.00	0.036	0.018				
9	GRYQQT/GRYRQT	0.107	0.036	0.071				
10	GRYRQT/GRYRQT	0.071	0.000	0.036				
11	GRYRQT/GRYQLT	0.071	0.036	0.054				
12	GRYHQT/GRYNQT	0.000	0.036	0.018				
13	GRYKQT/GRYQLT	0.071	0.00	0.036				
14	GRYQQT/GRYKQT	0.036	0.036	0.036				
15	GRYQLT/GRYQLT	0.071	0.071	0.071				

Alleles and genotypes associated with susceptibility/resistance to scrapie are underlined.

In goats, G_22_P_63_H_143_P_240 _allele was maximally found with an overall frequency of 0.527, followed by the wild-type allele G_22_P_63_H_143_S_240 _that represented 30.4% of the alleles (Table 1). The maximally detected genotype was G_22_P_63_R_143_P_240_/G_22_P_63_H_143_P_240 _(0.304) followed by G_22_P_63_H_143_P_240_/G_22_P_63_H_143_P_240 _(0.250). Two alleles C_22_P_63_H_143_P_240 _and G_22_L_63_H_143_P_240 _carried the new amino acid polymorphisms G22C (ggc22tgc) and P63L (ccc63ctc). Both G22C and P63L were detected in heterozygous state. The three amino acid polymorphisms (22C, 63L and 143R) were found linked with 240P. Three previously known silent mutations (ccg42cca - P42P, ggc83gga - G83G and agc138agt - S138S) were also detected. Both 42a and 138t were linked with 240P in most of animals. A few goats with linkage of 42g or 138c with 240S were also found.

## Discussion

The European sheep and goat husbandry has suffered huge economic losses due to scrapie outbreaks. Scrapie like other prion diseases has equal chance of being transmitted within and among species, depending on the PrP gene sequence [5,6]. The advent of modern gene sequence technologies have now paved the path for breeders to make sheep and goats resistant against upsetting disorders including scrapie by helping them in determining the allele frequencies, which is a prerequisite to devise an appropriate breeding plan.

Resistance to natural or experimental PrP^Sc ^in sheep scrapie is reported to be controlled by M112T, A136V, M137T, I142K, R154H, P168L, Q171R/H and N176K polymorphisms of the PrP gene [10,11,25-30]. However, only A136V, R154H and Q171R/H have been targeted for selective breeding against scrapie susceptibility based on their accepted involvement in the modulation of resistance to the disease [9]. The PrP gene carrying Q171R polymorphism confers the highest resistance to sheep scrapie. Conversely, the presence of A136V polymorphism in the PrP gene makes sheep highly susceptible to the disease. In the present study, no sheep was found carrying the A136V polymorphism. This polymorphism has also not been detected in our previous studies involving nine Pakistani sheep breeds [31,32]. Moreover, F141L that is a risk factor for atypical scrapie [33] was absent from the PrP gene of the studied sheep. R154H that is another risk factor for atypical scrapie [33,34] but is known to confer resistance to typical scrapie in both sheep and goats [10,18], was also not detected for the studied sheep and goats. Except Q171R/H, no other polymorphism associated with sheep scrapie was detected for Damani and Hashtnagri (Table 1). But the same as in our previous studies [33,34], its frequency (0.107) is lower raising the scrapie risk in the studied sheep breeds.

Amongst caprine PrP polymorphisms I142M, H143R, N146S/D, R154H, R211Q and Q222K associated with resistance to scrapie [13-18], only H143R was detected for the studied goats. This polymorphism has also been detected in other Pakistani goat breeds like Pak Angora, Teddy and Beetal [35]. Like sheep, the lower frequency of H143R may raise the scrapie risk in the studied goat breeds. The only polymorphism that is common in goats both from Pakistan [35,36] as well as other countries of the world [23] is S240P. As was the case in this study, this polymorphism is often found linked with other amino acid polymorphisms in the caprine PrP gene [9].

Due to species barrier conferred by sequence variation, the rare polymorphisms in the PrP gene may also be useful in protecting animals against PrP^Sc ^infection. In the present study, two new and rare PrP polymorphisms were detected in each of sheep (Q171N and T191I, accession # GQ497224 and GQ497223, respectively) and goats (G22C and P63L, accession # GQ497228 and GQ497222, respectively) (Table 1).

## Competing interests

The authors declare that they have no competing interests.

## Authors' contributions

AH carried out experiments, performed sequence alignments and participated in writing the manuscript and data analysis. MEB conceived, designed and supervised the study. MI analyzed the data and wrote the manuscript. IUH and MMJ coordinated the study. All authors read and approved the manuscript.
